# Hypothermia in trauma victims at first arrival of ambulance personnel: an observational study with assessment of risk factors

**DOI:** 10.1186/s13049-017-0349-1

**Published:** 2017-04-24

**Authors:** Frédéric Lapostolle, James Couvreur, François Xavier Koch, Dominique Savary, Armelle Alhéritière, Michel Galinski, Jean-Luc Sebbah, Karim Tazarourte, Frédéric Adnet

**Affiliations:** 1 SAMU 93, Unité de recherche-enseignement-qualité, Avicenne125, rue de Stalingrad, 93009 Bobigny, France; 2 Université Paris 13, Sorbonne Paris Cité, 93000 Bobigny, France; 3 Pôle Urgence-SAMU-SMUR, Hôpital Michallon, La Tronche, France; 4 SAMU 74, avenue de l’hôpital, Pringy, France; 5 SMUR, Centre hospitalier de Gonesse, Gonesse, France; 6 Pôle urgence-réanimation-SAMU 77, Centre hospitalier Marc Jacquet, Melun, France

**Keywords:** Trauma, Body temperature, Prehospital settings

## Abstract

**Background:**

Hypothermia is common in trauma victims and is associated with increased mortality, however its causes are little known. The objective of this study was to identify the risk factors associated with hypothermia in prehospital management of trauma victims.

**Methods:**

This was an ancillary analysis of data recorded in the HypoTraum study, a prospective multicenter study conducted by the emergency medical services (EMS) of 8 hospitals in France. Inclusion criteria were: trauma victim, age over 18 years, and victim receiving prehospital care from an EMS team and transported to hospital by the EMS team in a medically equipped mobile intensive care unit. The following data were recorded: victim demographics, circumstances of the trauma, environmental factors, patient presentation, clinical data and time from accident to EMS arrival. Independent risk factors for hypothermia were analyzed in a multivariate logistic regression model.

**Results:**

A total of 461 trauma patients were included in the study. Road traffic accidents (*N* = 261; 57%) and falls (*N* = 65; 14%) were the main causes of trauma. Hypothermia (<35 °C) was present in 136/461 cases (29%). Independent factors significantly associated with the presence of hypothermia were: a low GCS (Odds Ratio (OR) = 0,87 ([0,81-0,92]; *p* < 0.0001), a low air temperature (*OR* = 0,93 [0,91-0,96]; *p* < 0.0001) and a wet patient (*OR* = 2,08 [1,08-4,00]; *p* = 0.03).

**Conclusion:**

The incidence of hypothermia was high on EMS arrival at the scene. Body temperature measurement and immediate thermal protection should be routine, and special attention should be given to patients who are wet.

**Level of evidence:**

Prospective, multicenter, open, observational study; Level IV.

## Background

Hypothermia is common in trauma victims. In several studies on severe trauma injury, hypothermia has been reported as occurring in up to two third of the patients [1]. Hypothermia is associated with aggravated injury and increased mortality [2, 3]. A body temperature below 35 °C is an independent risk factor of mortality in trauma victims [4, 5]. Hypothermia induced coagulopathy and cardiovascular, neurological, renal and hematologic effects, all contribute to increased morbidity and mortality [1, 6]. In addition, the lower the initial body temperature, the greater the incidence of hypothermia. Controlling body temperature is thus a priority in the early management of trauma victims [1]. Dedicated guidelines have been recently published [1]. Yet, hypothermia is underdiagnosed and undertreated, particularly during initial management of severe trauma, and only a few studies have focused on prehospital management of trauma victims’ body temperature [1].

The HypoTraum study has shown that risk factors predictive of hypothermia on arrival at the hospital emergency department (ED) relate to the victim’s environment, the severity of the injury, and prehospital management, especially during transport to hospital [7]. On the other hand, circumstances leading hypothermia, from trauma onset to rescue team arrival, remain less clear. Such information should help to optimize body temperature control, and improve management and final outcome.

The aim of the present study was to investigate the risk factors associated with hypothermia, not on arrival at hospital, but at the time of arrival of the emergency medical services (EMS) team at the scene of the accident.

## Methods

### Study design

The HypoTraum study was a prospective, multicenter, open, observational study to determine the risk factors for hypothermia on arrival at hospital. Full details of the methods have been previously published [7].

### Setting

In France, all emergency medical calls connect to dispatching centers called “SAMU” [8]. All calls are handled by an emergency physician dispatcher. Among different interventional means at his disposal, the dispatcher can send to the field, when required, mobile intensive care units (MICU), spread among operational centers over each French administrative region. Each MICU is staffed, at least, by an emergency physician and by a specifically trained nurse and driver. Depending on center location, a medical student can be added to the team. MICUs are capable of performing most techniques and therapies available in the emergency department; including venous access, airway management, chest drain insertion, damage control care, blood volume expansion and anesthetics and intropic/vasopressor support. Other devices are increasingly embedded, as blood sample analysers or portable ultrasound machines (for a complete presentation of EMS organization in France, see reference [8]).

### Participants

Patients were included by MICUs from eight French operational centers. Inclusion criteria were: trauma victim, age over 18 years, and victim receiving prehospital care from an EMS team and transported to hospital by the EMS team in a MICU.

### Variables and data source

The following data were recorded: patient demographics and morphological traits (age, sex, weight, and height), circumstances of the trauma (type of accident, date, time, place), environmental conditions (air and ground temperatures using a non-contact IR thermometer, TN1 Nonfumo flue systems®, High Wycombe, UK), wind speed with an anemometer (La Crosse Technology®, Geispolsheim, France), and rain, victim presentation (whether trapped, seated or lying down, unclothed, wet, or protected by a blanket), clinical data (nature of trauma, Glasgow Coma Score (GCS), systolic blood pressure, heart and respiratory rates, oxygen saturation (and oxygen delivery)), Revised Trauma Score (RTS), epitympanic temperature and time from accident to EMS arrival. Rain and wetness were subjectively evaluated by the physician, in the field. To characterize patient evolution, body temperature at arrival at hospital was also recorded and compared to initial temperature.

**Fig. 1:**
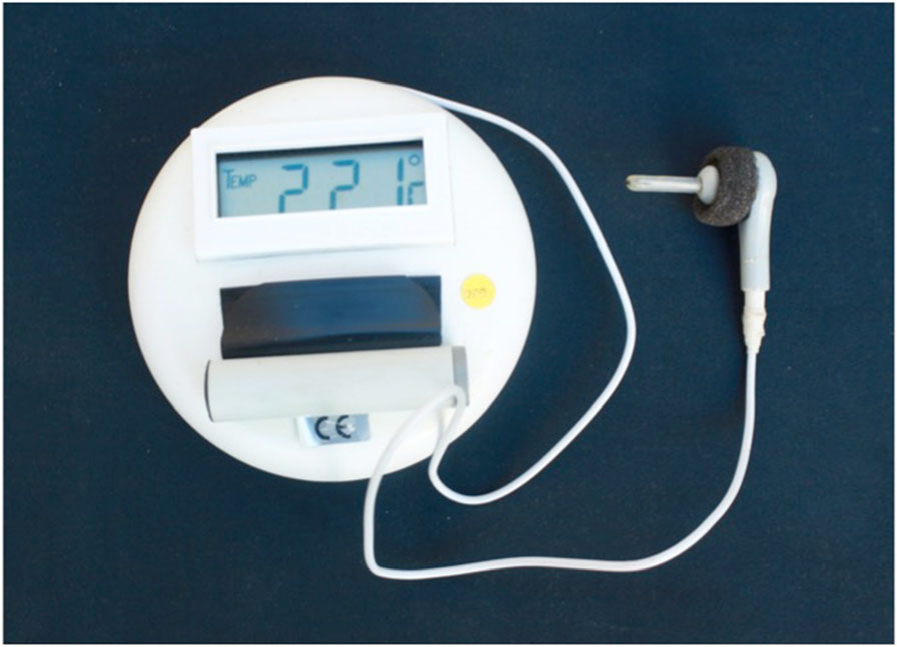
Metraux® tympanic thermometer (epitympanic probe and isolating cushion)

Epitympanic temperature was obtained by using a Métraux® tympanic thermometer (Crissier, Switzerland) [9, 10]. This device includes an 18 mm (epitympanic) probe and an isolating cushion (Fig. 1). According to standardized procedure, it was carefully introduced in the outer ear canal and held in place by adhesive bandage in order to obtain optimal isolation. The first stable temperature was recorded.

Data were prospectively recorded, in the field, by the emergency physician or the nurse on a dedicated paper form. The first measurement was recorded for each of the criteria.

The main endpoint was body temperature as measured by the EMS team on arrival at the accident scene.

Hypothermia was defined as a body temperature < 35 °C [1, 3–5, 7, 11–13].

### Statistical analysis

Results are expressed as medians with interquartile ranges. Quantitative and qualitative data were compared by the Mann–Whitney and Chi-square tests, respectively. *P* values of 0.05 or less were considered significant. Factors with a *p* value <0.2 in univariate analyses were selected for inclusion in a multivariate logistic regression model (Statview 5.0, SAS Institute, Cary, NC, USA). Odds ratios (ORs) were calculated.

The study was approved by the local Ethics Committee (Committee for the Protection of Persons – CPP Ile de France X, Hôpital Robert Ballanger, Aulnay-sous-bois, France).

## Results

### Participants

Between January 1, 2004, and November 10, 2007, 461 trauma victims managed by the EMS services of 8 hospitals were included in the study. The median age of the patients was 34 (23–46) years, 334 (72%) male and 127 (28%) female. Road accidents (*N* = 261; 57%) and falls (*N* = 65; 14%) were the main causes of trauma. Median air temperature at inclusion was 17.0 (10.3-22.3)°C. The median time between accident and body temperature measurement was 30 (24–45) min. Hypothermia (<35 °C) was present in 136/461 (29%) victims on arrival of the EMS mobile unit at the accident scene.

### Main results

A comparison of trauma victims with and without hypothermia on EMS arrival is given in Tables 1 and 2. Victim characteristics and environmental factors associated with the presence of hypothermia (*p* < 0.2) were by decreasing rank order: air and ground temperature, body mass index (BMI) and body weight, daytime, indoor accident, type of accident, season of the year, and rain (Table 1). Factors associated with victim presentation and clinical characteristics were GCS and RTS, whether victim wet, on the ground or trapped, systolic blood pressure, and heart rate (Table 2). After entry of these factors into a multivariate analysis, only a low GCS, a low air temperature, and a wet patient proved to be independent factors associated with hypothermia (Table 3).

**Table 1:** Trauma victim demographics and environmental conditions at the scene of the accident

	Hypothermia	No hypothermia	*p*
	*N* = 136	*N* = 325	
Demographics and traits			
Age (yr)	33 (23–47)	34 (23–46)	0.9
Male - n (%)	105 (78)	229 (71)	0.1
Body weight (kg)	70 (60–80)	75 (65–80)	0.02
Height (cm)	173 (168–180)	174 (165–180)	0.5
Body Mass Index (kg/m^2^)	23.5 (21.1-26.1)	24.7 (22.1-27.7)	0.01
Environmental conditions – n (%)			
Daytime - n (%)	81 (63)	226 (72)	0.05
Season - n (%)			
Winter	47 (35)	87 (28)	0.09
Spring	34 (25)	76 (23)	
Summer	25 (18)	93 (29)	
Autumn	30 (22)	69 (21)	
Indoors - n (%)	18 (13)	70 (22)	0.06
Air temperature (°C)	12.3 (7.6-18.2)	18.6 (12.1-23.6)	<0.0001
Ground temperature (°C)	12.1 (7.1-18.2)	19.1 (11.7-23.6)	<0.0001
Windy - n (%)	12 (9)	25 (8)	0.7
Rain - n (%)	20 (15)	31 (10)	0.1
Type of accident – n (%)			
Road accident	71 (52)	190 (58)	0.07
Fall	49 (36)	95 (29)	
Weapon (gun or knife)	5 (4)	15 (5)	
Other	11 (8)	25 (8)	

Results are expressed as numbers with percentages or as medians with interquartile ranges

**Table 2:** Trauma victim presentation and clinical examination on arrival of EMS team at the scene of the accident

	Hypothermia	No hypothermia	*p*
	*N* = 136	*N* = 325	
Presentation			
Trapped - n (%)	24 (18)	36 (11)	0.07
Time from accident to body temperature measurement (min)	30 (22–43)	30 (24–45)	0.8
Position - n (%)			
Seated	22 (16)	49 (15)	0.8
Lying down	114 (84)	272 (85)	
On the ground - n (%)	91 (67)	184 (57)	0.05
Unclothed - n (%)	41 (30)	107 (33)	0.6
Wet - n (%)	26 (19)	23 (7)	0.0004
Covered by blanket - n (%)	75 (55)	157 (48)	0.2
Shivering - n (%)	25 (18)	54 (17)	0.7
Clinical examination			
Glasgow Coma Score (GCS)	15 (9–15)	15 (15–15)	<0.0001
Systolic blood pressure (mmHg)	123 (108–140)	126 (110–140)	0.1
Heart rate (bpm)	84 (70–101)	90 (75–100)	0.1
Respiratory rate (breaths/min)	18 (16–22)	18 (16–22)	0.8
Pulse oxymetry (%)	99 (97–100)	99 (97–100)	0.8
Nature of injury – n (%)			
- head	71 (52)	0 5)	0.2
- chest	39 (29)	0 1)	0.7
- abdomen	17 (12)	42 13)	1
- hip	22 (16)	54 17)	1
- limbs	59 (43)	128 (39)	0.5
Revised Trauma Score (RTS)	11 (10–11)	11 (11–11)	<0.0001
Status on arrival at hospital ED - n (%)	62 (13)	399 (87)	

Results are expressed as numbers with percentages or as medians with interquartile ranges

RTS = GCS + systolic blood pressure + respiratory rate

**Table 3:** Independent factors associated with presence of hypothermia in multivariate analysis (*N* = 450)

Factor	Odds Ratio [95% CI]	*p*
Glasgow Coma Score	0,87 [0,81-0,92]	<0.0001
Air temperature	0,93 [0,91-0,96]	<0.0001
Wet patient	2,08 [1,08-4,00]	0.03

*CI* confidence interval

**Fig. 2:**
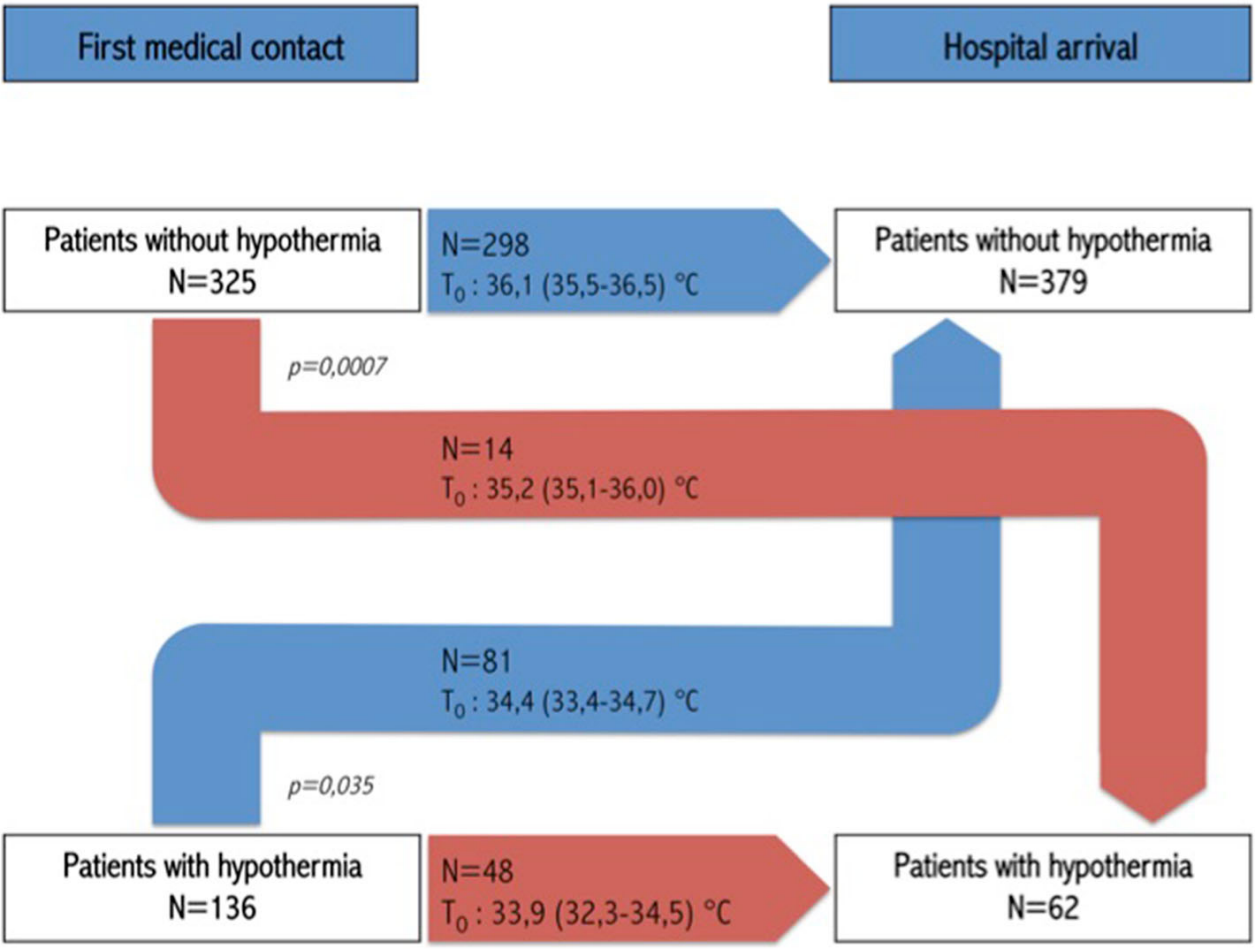
Distribution of the patient according to the presence of hypothermia at the time of the first medical contact or hospital arrival. To indicates the initial median (interquartile) temperature

Median time between first medical contact and arrival at hospital was 60 (46–80) min. In the group of patients without hypothermia on EMS arrival on-scene, only 14 (4%) patients had hypothermia when arriving at hospital. While in the group of patients with hypothermia on EMS arrival on-scene, 48 (32%) had persisting hypothermia. Initial median temperatures between groups were significantly different (*p* < 0,0001). Results are detailed in Fig. 2.

## Discussion

The prevalence of hypothermia was high (29%) on EMS arrival at the scene of the accident and especially noteworthy as patient body temperature was measured after a median delay of only 30 min after the accident. Because of this high incidence and because hypothermia in an independent risk factor of mortality in trauma victims, we recommend the routine early measurement and monitoring of body temperature in all trauma victims.

In this prehospital setting, three independent factors predictive of hyperthermia were identified at the scene of the accident: a low level of consciousness of the victim as given by the GCS, a low air temperature (median 17 °C in this study), and a wet victim. The risk of hypothermia was at least two-fold higher in wet patients, even if these patients accounted for only 11% of the overall population (49/461). A patient who is drenched should benefit from special measures. Usually this means being undressed, dried, and provided with thermal protection. These actions are particularly important as there are no specific interventions that can compensate for injury severity (as given here by the RTS). Undressing, however, should be reserved for wet patients only as it becomes an independent risk factor of hypothermia on arrival at hospital [7]. Blankets were frequently used before MICU arrival and moreover after (74%) [7]. In contrast, active warming was very rarely used during management by the MICU (3%) [7].

The incidence of hypothermia recorded in our earlier study on arrival at the hospital ED was considerably lower (14% vs 29%). This may have been due in part to patient warming by the medical team during transport to hospital in 78% of cases, even if harmful interventions such as infusion of unwarmed fluid may have taken place [7]. In the present study, only 50% of patients (232/461) benefitted from warming (covered by blanket) before EMS arrival and hypothermia was controlled in fewer than 50% of cases whatever the measures taken by the medical team.

The main risk factors for hypothermia on arrival of trauma victims at the hospital ED were severity of injury (especially head injury) and certain aspects of medical care (e.g. orotracheal intubation) [7]. However, the nature of the injury was not an independent risk factor for hypothermia in an early prehospital setting, maybe because the interval between the accident and body temperature measurement was too short for the expression of all adverse effects.

Initial measurement of body temperature is crucial in the initial assessment of trauma patients. We strongly feel that body temperature should be considered as a vital parameter along with blood pressure and heart rate. Continuous monitoring should be performed where possible, particularly in severely injured patients. Only one non invasive device allowed pre-hospital was validated for continuous monitoring when we started this study [9]. A few more devices are now available [1]. This underlines the special attention currently being paid to temperature management in trauma patients.

Precocious, prehospital hypothermia diagnosis is the key to optimal hypothermia management. In this study, hypothermia was corrected (or avoided) before hospital arrival in two thirds of patients. As the Hypotraum study was conducted in France, involvement of an emergency physician in the prehospital setting and relatively short transportation times may have contributed to hypothermia management. First-aid rescuers must also be trained to manage temperature, and long transportation times need to be used to correct hypothermia.

The strength of our study was the exploration of a wide variety of factors (environmental and other) poorly documented in an early prehospital setting [3, 13–15]. Data collection was facilitated by the way the French EMS system is organized. In France, mobile intensive care units with an emergency physician aboard are sent out to accident scenes. However, the system was also a potential limitation of our study as, in the case of the most serious accidents, the fire brigade may have preceded the medical team on scene, commencing first warming measures, such as blankets, before MICU arrival and initial assessment. Conversely, patients most in need of medical management may have had protracted periods outside on the ground before they could be moved. In contrast less severely injured patients could be managed without MICU, however, such minor trauma patients are not at high risk of hypothermia. Nevertheless, it is unlikely that the determinants of the onset of hypothermia on EMS arrival are influenced by the EMS system. Furthermore, the time from accident to MICU arrival, the position of the patient, and the presence of a blanket were not significantly different between patients with and without hypothermia. Finally, it cannot be excluded that the respective impact of ambient temperature, wetness and patient’s injury severity on body temperature could be different in other locations.

## Conclusion

Early preventive or corrective measures should help reduce the incidence of hypothermia in a prehospital setting. These measures should be initiated as soon as EMS arrive at the accident scene. Body temperature measurement and immediate thermal protection should be routine, and special attention should be given to patients who are wet. These measures should be followed by optimal patient management during transport to hospital (warming of infusion fluids and ambulance heating) as previously documented.

## Abbreviations

BMI: Body mass index
CPP: Committee for the Protection of Persons
ED: Emergency Department
EMS: Emergency medical services
GCS: Glasgow coma score
OR: Odds ratio
RTS: Revised trauma score
